# (*S*)-*tert*-Butyl 3-(3-phenyl-1,2,4-oxa­diazol-5-yl)piperidine-1-carboxyl­ate

**DOI:** 10.1107/S1600536810018714

**Published:** 2010-06-05

**Authors:** Lin Liu, Guangxin Xia, Xuejun Liu, Jieshu Xie, Jingkang Shen

**Affiliations:** a Shanghai Institute of Materia Medica, Shanghai Institutes for Biological Sciences, Chinese Academy of Sciences, 555 Zuchongzhi Road, Shanghai 201203, People’s Republic of China; b Central Research Institute, Shanghai Pharmaceutical (Group) Co. Ltd, 555 Zuchongzhi Road, Shanghai 201203, People’s Republic of China

## Abstract

The title compound, C_18_H_23_N_3_O_3_, crystallized with two independent mol­ecules (*A* and *B*) in the asymmetric unit. The phenyl ring and the 1,2,4-oxadiazole ring are inclined to one another by 19.9 (3)° in mol­ecule *A* and 7.3 (3)° in mol­ecule *B*. The absolute structure of the title compound was referred to the transfered chiral center (*S*) of one of the starting reacta­nts. In the crystal, *A* mol­ecules are linked by C—H⋯N inter­actions involving the two oxadiazole N atoms.

## Related literature

For the oxadiazole nucleus as a core structural unit of various muscarinic agonists, see: Orlek & Blaney (1991 ▶). For benzodiazepine receptor partial agonists, see: Watjen & Baker (1989 ▶). For dopamine transporters, see: Gray & Abrahm (1993 ▶). For 5-HT agonists, see: Swain & Baker (1991 ▶). For inhibitors of HIV, see: Matthew *et al.* (2007 ▶). For GABAA receptor agonists, see: Michaela & Holger (2008 ▶). For bond-length data, see: Allen *et al.* (1987 ▶).
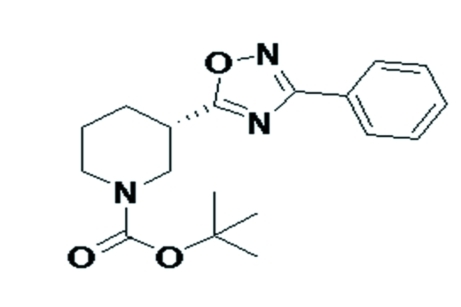


## Experimental

### 

#### Crystal data

C_18_H_23_N_3_O_3_
*M* *_r_* = 329.39
Monoclinic, 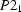

*a* = 6.464 (3) Å
*b* = 15.515 (8) Å
*c* = 17.847 (9) Å
β = 99.880 (7)°
*V* = 1763.2 (15) Å^3^
*Z* = 4
Mo *K*α radiation
μ = 0.09 mm^−1^
*T* = 293 K
0.24 × 0.15 × 0.12 mm

#### Data collection

Bruker SMART CCD area-detector diffractometer
Absorption correction: multi-scan (*SADABS*; Sheldrick, 2008 ▶) *T*_min_ = 0.980, *T*_max_ = 0.990
7378 measured reflections
3258 independent reflections
2520 reflections with *I* > 2σ(*I*)
*R*_int_ = 0.073

#### Refinement

*R*[*F* ^2^ > 2σ(*F* ^2^)] = 0.073
*wR*(*F* ^2^) = 0.198
*S* = 1.01
3258 reflections
440 parameters
1 restraint
H-atom parameters constrained
Δρ_max_ = 0.29 e Å^−3^
Δρ_min_ = −0.44 e Å^−3^

### 

Data collection: *SMART* (Bruker, 2007 ▶); cell refinement: *SAINT* (Bruker, 2007 ▶); data reduction: *SAINT*; program(s) used to solve structure: *SHELXS97* (Sheldrick, 2008 ▶); program(s) used to refine structure: *SHELXL97* (Sheldrick, 2008 ▶); molecular graphics: *PLATON* (Spek, 2009 ▶); software used to prepare material for publication: *SHELXL97* and *PLATON*.

## Supplementary Material

Crystal structure: contains datablocks I, global. DOI: 10.1107/S1600536810018714/su2175sup1.cif

Structure factors: contains datablocks I. DOI: 10.1107/S1600536810018714/su2175Isup2.hkl

Additional supplementary materials:  crystallographic information; 3D view; checkCIF report

## Figures and Tables

**Table 1:** Hydrogen-bond geometry (Å, °)

*D*—H⋯*A*	*D*—H	H⋯*A*	*D*⋯*A*	*D*—H⋯*A*
C13—H13*A*⋯N2^i^	0.97	2.61	3.352 (7)	133
C18—H18*A*⋯N1^i^	0.96	2.61	3.490 (9)	153

Symmetry code: (i) 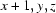.

**Fig. 1.**
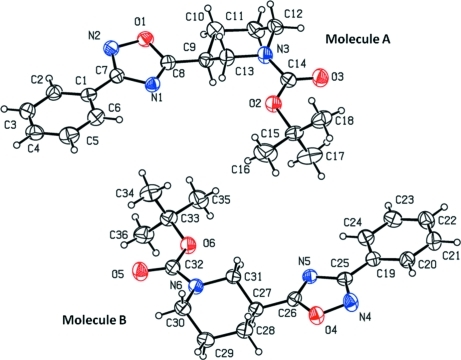
The molecular structure of the two independent molecules (A and B) of the title compound, with atom labels and 30% probability displacement ellipsoids.

**Fig. 2.**
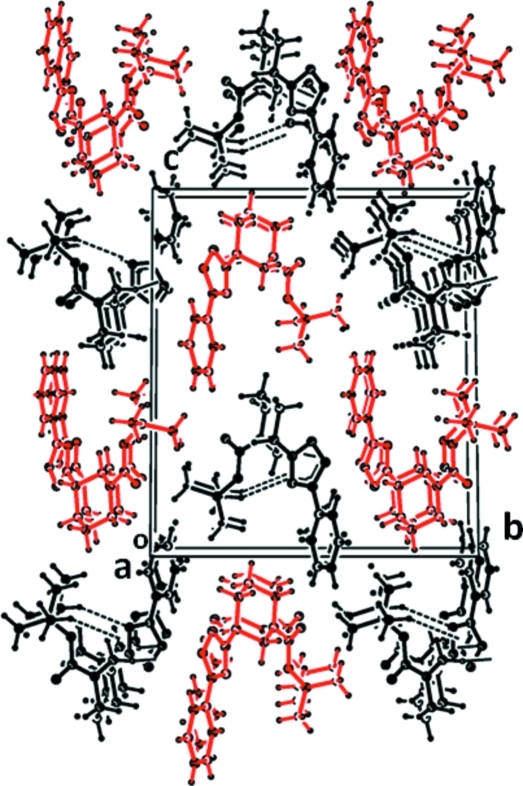
A view along the a-axis of the crystal packing of the title compound (Molecule A is black; Molecule B is red; C-H···N interactions are shown as dotted lines; see Table 1 for details).

## supplementary crystallographic information

### Comment

The oxadiazole nucleus is a well studied pharmacophoric scaffold that has emerged as a core structural unit of various muscarinic agonists (Orlek & Blaney, 1991), benzodiazepine receptor partial agonists (Watjen & Baker, 1989), dopamine transporters (Gray & Abrahm, 1993), 5-HT agonists (Swain & Baker, 1991), inhibitors of HIV (Matthew, 2007), and GABAA receptor agonists (Michaela & Holger,2008). Among the oxadiazoles, 1,2,4-oxadiazole derivatives have gained importance in medicinal chemistry. The interest in five-membered systems containing one oxygen and two nitrogen atoms (positions 1, 2, and 4) is due to the occurrence of 1,2,4-oxadiazoles in biological activite compounds and natural products. In spite of extensive investigations, there are few studies on the crystal structures of oxadiazol-piperidines. Herein, we report on the crystal structure of the title compound, a new oxadiazol-piperidine. It can be reacted with acid, sulfonlchloride and chloride, followed by deprotection of the protective group, to give many usefull compounds.

The title compound crystallized in the chiral monoclinic space group P2_1_, with two independent molecules (A and B) in the asymmetric unit (Fig. 1). It was obtained from a chiral source, hence its absolute structure, (S), was confirmed by the transfered chiral center; atom C9 in molecule A and atom C27 in molecule B (Fig. 1). The bond distances in the two molecules are very similar and close to normal values (Allen *et al.*, 1987). The two molecules differ in the orientation of the phenyl ring with respect to the oxadiazole mean plane. In molecule A this dihedral angle is 19.9 (3)°, while in molecule B it is only 7.3 (3)°. In both molecules the piperidine ring has a chair conformation.

In the crystal symmetry related A molecules are linked via C-H···N interactions (see Table 1 and Fig. 2 for details).

### Experimental

A suspension of hydroxylamine hydrochloride (4.09 g), potassium carbonate (2.76 g), benzonitrile (1.03 g) in absolute ethanol (200 mL) was heated at reflux for 10 h. After the reaction was completed, monitored by TLC, the mixture was cooled, filtered to remove inorganic salts, and concentrated under vacuum. The residue was purified by column chromatography, by use of a gradient elution of dichloromethane to 40% acetone in dichloromethane, to give (E)-N-hydroxybenzamidine (1.36g). ^1^H NMR (300 MHz, DMSO-d6): 9.59 (s, 1H), 7.62-7.67 (m, 2H), 7.32-7.37 (m, 3H), 5.77 (s, 2H); EI (M+) 136 A mixture of (S)-1-(tert-butoxycarbonyl)piperidine-3-carboxylic acid (1.15 g) in absolute THF (50 mL), isobutyl carbonochloridate (5ml) and trimethylamine (2ml) were mixted together and stirred for 30min at rt, followed by slow dropwise addition of (E)-N-Hydroxy-benzamidine (1.36g) in THF (15mL). After the reaction was completed, monitored by TLC, the mixture was injected into n-Bu4NF (1 M in THF, 3 mL), warmed to reflux and was stirred for 24 h. After this reaction was completed, monitored by TLC, the mixture was poured into EtOAc and washed with water and brine. The organic layer was dried (MgSO4) and concentrated in vacuo. The residue was purified by column chromatography by use of a gradient elution of EtOAc/hexanes. The material was crystallized from EtOH to give the title compound as a white solid. Colourless-rod-like crystals, suitable for X-ray analysis, were obtained by recrystallization from EtOH. ^1^H NMR (300MHz, CDCl3): 8.05,-8.08 (m, 2H), 7.43-7.48 (m, 3H), 3.95 (d, 1H, J=8.4 Hz), 3.12-3.17 (m, 1H), 3.98 (t, 1H, J=12.6 Hz), 2.42 (d, 1H, J=12.6 Hz), 1.86 (t, 2H, J=12.6 Hz), 1.55-1.65 (m, 1H), 1.45 (s, 9H), 0.83-0.92 (m, 2H); ESI (M++23) 352.

### Refinement

In the final cycles of refinement, in the absence of significant anomalous scattering effects, 4120 Friedel pairs were merged and Δf " set to zero. The H-atoms could all be located in difference Fourier maps. In the final cycles of refinment they were placed in calculated positions and treated as riding atoms: C—H 0.93, 0.96, 0.97 and 0.98 Å, for H-methine, H-methyl, H-methylene and H-aromtic, respectively, with U_iso_(H) = k × U_eq_(C), where k = 1.5 for H-methyl and = 1.2 for all other H-atoms.

### Crystal data

**Table:**

C_18_H_23_N_3_O_3_	*F*(000) = 704
*M**_r_* = 329.39	*D*_x_ = 1.241 Mg m^−^^3^
Monoclinic, *P*2_1_	Mo *K*α radiation, λ = 0.71073 Å
Hall symbol: P 2yb	Cell parameters from 818 reflections
*a* = 6.464 (3) Å	θ = 2.7–23.3°
*b* = 15.515 (8) Å	µ = 0.09 mm^−^^1^
*c* = 17.847 (9) Å	*T* = 293 K
β = 99.880 (7)°	Rod, colourless
*V* = 1763.2 (15) Å^3^	0.24 × 0.15 × 0.12 mm
*Z* = 4	

### Data collection

**Table:**

Bruker SMART CCD area-detector diffractometer	3258 independent reflections
Radiation source: fine-focus sealed tube	2520 reflections with *I* > 2σ(*I*)
graphite	*R*_int_ = 0.073
phi and ω scans	θ_max_ = 25.1°, θ_min_ = 1.8°
Absorption correction: multi-scan (*SADABS*; Sheldrick, 2008)	*h* = −7→7
*T*_min_ = 0.980, *T*_max_ = 0.990	*k* = −18→16
7378 measured reflections	*l* = −21→14

### Refinement

**Table:**

Refinement on *F*^2^	Secondary atom site location: difference Fourier map
Least-squares matrix: full	Hydrogen site location: inferred from neighbouring sites
*R*[*F*^2^ > 2σ(*F*^2^)] = 0.073	H-atom parameters constrained
*wR*(*F*^2^) = 0.198	*w* = 1/[σ^2^(*F*_o_^2^) + (0.1416*P*)^2^] where *P* = (*F*_o_^2^ + 2*F*_c_^2^)/3
*S* = 1.01	(Δ/σ)_max_ < 0.001
3258 reflections	Δρ_max_ = 0.29 e Å^−^^3^
440 parameters	Δρ_min_ = −0.44 e Å^−^^3^
1 restraint	Extinction correction: *SHELXL97* (Sheldrick, 2008), Fc^*^=kFc[1+0.001xFc^2^λ^3^/sin(2θ)]^-1/4^
Primary atom site location: structure-invariant direct methods	Extinction coefficient: 0.013 (4)

### Special details

**Table:**

Geometry. Bond distances, angles etc. have been calculated using the rounded fractional coordinates. All su's are estimated from the variances of the (full) variance-covariance matrix. The cell esds are taken into account in the estimation of distances, angles and torsion angles
Refinement. Refinement of F^2^ against ALL reflections. The weighted R-factor wR and goodness of fit S are based on F^2^, conventional R-factors R are based on F, with F set to zero for negative F^2^. The threshold expression of F^2^ > 2sigma(F^2^) is used only for calculating R-factors(gt) etc. and is not relevant to the choice of reflections for refinement. R-factors based on F^2^ are statistically about twice as large as those based on F, and R- factors based on ALL data will be even larger.

### Fractional atomic coordinates and isotropic or equivalent isotropic displacement parameters (Å^2^)

**Table:**

	*x*	*y*	*z*	*U*_iso_*/*U*_eq_	
O1	0.4355 (6)	0.9886 (3)	0.68689 (18)	0.0605 (12)	
O2	1.1738 (6)	0.7663 (3)	0.79331 (18)	0.0644 (15)	
O3	1.2986 (7)	0.7271 (3)	0.6870 (2)	0.0747 (16)	
N1	0.5574 (6)	0.9308 (3)	0.7976 (2)	0.0536 (14)	
N2	0.3265 (7)	1.0282 (3)	0.7402 (2)	0.0603 (17)	
N3	1.0660 (7)	0.8361 (3)	0.6855 (2)	0.0626 (16)	
C1	0.3287 (8)	1.0093 (3)	0.8751 (3)	0.0524 (19)	
C2	0.1341 (9)	1.0467 (4)	0.8740 (3)	0.062 (2)	
C3	0.0626 (11)	1.0639 (4)	0.9411 (3)	0.070 (2)	
C4	0.1837 (11)	1.0425 (4)	1.0097 (3)	0.072 (2)	
C5	0.3769 (12)	1.0042 (4)	1.0113 (3)	0.079 (2)	
C6	0.4488 (9)	0.9868 (4)	0.9447 (3)	0.067 (2)	
C7	0.4026 (7)	0.9904 (3)	0.8048 (3)	0.0496 (17)	
C8	0.5670 (7)	0.9310 (3)	0.7267 (3)	0.0496 (17)	
C9	0.6996 (8)	0.8775 (4)	0.6851 (3)	0.0529 (17)	
C10	0.6740 (9)	0.8942 (5)	0.6013 (3)	0.068 (2)	
C11	0.8260 (9)	0.8384 (5)	0.5657 (3)	0.067 (2)	
C12	1.0481 (9)	0.8513 (4)	0.6046 (3)	0.061 (2)	
C13	0.9262 (8)	0.8882 (4)	0.7234 (2)	0.0584 (18)	
C14	1.1864 (8)	0.7715 (4)	0.7210 (3)	0.0542 (17)	
C15	1.2965 (10)	0.7014 (4)	0.8439 (3)	0.067 (2)	
C16	1.2285 (14)	0.7220 (6)	0.9186 (3)	0.106 (3)	
C17	1.2262 (15)	0.6118 (5)	0.8167 (4)	0.102 (3)	
C18	1.5243 (11)	0.7145 (5)	0.8480 (4)	0.086 (3)	
O4	0.6093 (6)	0.1946 (3)	0.82130 (18)	0.0652 (13)	
O5	−0.2882 (7)	0.4596 (3)	0.8012 (2)	0.0755 (16)	
O6	−0.1493 (6)	0.4210 (3)	0.69793 (18)	0.0678 (15)	
N4	0.7414 (7)	0.1666 (4)	0.7705 (2)	0.0672 (19)	
N5	0.4557 (7)	0.2334 (3)	0.7072 (2)	0.0514 (14)	
N6	0.0024 (7)	0.3759 (3)	0.8124 (2)	0.0583 (15)	
C19	0.7234 (8)	0.1768 (3)	0.6348 (3)	0.0502 (17)	
C20	0.9211 (9)	0.1437 (4)	0.6355 (3)	0.0614 (17)	
C21	0.9925 (10)	0.1274 (4)	0.5684 (3)	0.072 (2)	
C22	0.8669 (11)	0.1471 (4)	0.4995 (3)	0.075 (3)	
C23	0.6724 (10)	0.1798 (4)	0.4977 (3)	0.070 (2)	
C24	0.5980 (9)	0.1963 (4)	0.5641 (3)	0.0617 (19)	
C25	0.6435 (8)	0.1934 (3)	0.7043 (2)	0.0489 (17)	
C26	0.4423 (8)	0.2318 (3)	0.7778 (3)	0.0500 (16)	
C27	0.2658 (9)	0.2633 (3)	0.8154 (3)	0.0514 (16)	
C28	0.3205 (9)	0.2705 (4)	0.9012 (3)	0.0626 (19)	
C29	0.1270 (10)	0.2999 (4)	0.9314 (3)	0.069 (2)	
C30	0.0361 (10)	0.3818 (4)	0.8946 (3)	0.0642 (19)	
C31	0.1823 (9)	0.3471 (4)	0.7790 (3)	0.0569 (19)	
C32	−0.1542 (9)	0.4222 (4)	0.7715 (3)	0.0570 (19)	
C33	−0.2846 (9)	0.4763 (4)	0.6425 (3)	0.0621 (19)	
C34	−0.2377 (13)	0.5693 (5)	0.6619 (4)	0.088 (3)	
C35	−0.2098 (11)	0.4529 (5)	0.5692 (3)	0.083 (3)	
C36	−0.5115 (10)	0.4536 (5)	0.6379 (4)	0.081 (3)	
H2A	0.05080	1.06050	0.82770	0.0740*	
H3A	−0.06750	1.08990	0.93990	0.0850*	
H4A	0.13540	1.05380	1.05490	0.0870*	
H5A	0.45900	0.99000	1.05770	0.0950*	
H6A	0.57800	0.95980	0.94620	0.0800*	
H9A	0.66130	0.81720	0.69160	0.0640*	
H10A	0.70080	0.95460	0.59260	0.0820*	
H10B	0.53080	0.88150	0.57760	0.0820*	
H11A	0.78820	0.77820	0.56920	0.0800*	
H11B	0.81450	0.85280	0.51230	0.0800*	
H12A	1.13950	0.81190	0.58350	0.0730*	
H12B	1.09210	0.90970	0.59590	0.0730*	
H13A	0.96590	0.94840	0.72190	0.0700*	
H13B	0.94070	0.87100	0.77630	0.0700*	
H16A	1.26510	0.78050	0.93250	0.1590*	
H16B	1.29790	0.68390	0.95720	0.1590*	
H16C	1.07920	0.71480	0.91360	0.1590*	
H17A	1.26240	0.60190	0.76740	0.1530*	
H17B	1.07690	0.60700	0.81350	0.1530*	
H17C	1.29500	0.56980	0.85190	0.1530*	
H18A	1.55570	0.77500	0.85240	0.1300*	
H18B	1.56650	0.69220	0.80270	0.1300*	
H18C	1.59900	0.68480	0.89160	0.1300*	
H20A	1.00750	0.13220	0.68170	0.0740*	
H21A	1.12460	0.10330	0.56930	0.0860*	
H22A	0.91670	0.13770	0.45430	0.0900*	
H23A	0.58780	0.19140	0.45120	0.0840*	
H24A	0.46540	0.22030	0.56240	0.0740*	
H27A	0.15230	0.22090	0.80430	0.0620*	
H28A	0.36690	0.21510	0.92300	0.0750*	
H28B	0.43330	0.31180	0.91510	0.0750*	
H29A	0.02150	0.25480	0.92260	0.0830*	
H29B	0.16320	0.30870	0.98580	0.0830*	
H30A	−0.09650	0.39390	0.91100	0.0770*	
H30B	0.13090	0.42930	0.91090	0.0770*	
H31A	0.29170	0.39050	0.78690	0.0680*	
H31B	0.13950	0.33890	0.72460	0.0680*	
H34A	−0.29080	0.58380	0.70740	0.1320*	
H34B	−0.30370	0.60510	0.62080	0.1320*	
H34C	−0.08860	0.57840	0.67010	0.1320*	
H35A	−0.06140	0.46290	0.57470	0.1240*	
H35B	−0.28190	0.48780	0.52850	0.1240*	
H35C	−0.23880	0.39320	0.55790	0.1240*	
H36A	−0.55900	0.47250	0.68330	0.1220*	
H36B	−0.52850	0.39230	0.63280	0.1220*	
H36C	−0.59270	0.48150	0.59450	0.1220*	

### Atomic displacement parameters (Å^2^)

**Table:**

	*U*^11^	*U*^22^	*U*^33^	*U*^12^	*U*^13^	*U*^23^
O1	0.058 (2)	0.070 (2)	0.052 (2)	0.0050 (18)	0.0049 (16)	0.0100 (17)
O2	0.071 (3)	0.081 (3)	0.0403 (19)	0.015 (2)	0.0071 (16)	0.0061 (17)
O3	0.083 (3)	0.089 (3)	0.053 (2)	0.020 (2)	0.0144 (18)	0.0008 (19)
N1	0.052 (3)	0.056 (2)	0.051 (2)	0.006 (2)	0.0039 (17)	0.0042 (19)
N2	0.052 (3)	0.069 (3)	0.060 (3)	0.008 (2)	0.0096 (19)	0.011 (2)
N3	0.066 (3)	0.085 (3)	0.036 (2)	0.019 (2)	0.0064 (17)	0.002 (2)
C1	0.060 (4)	0.046 (3)	0.050 (3)	−0.004 (2)	0.006 (2)	0.003 (2)
C2	0.055 (4)	0.055 (3)	0.073 (4)	−0.002 (3)	0.007 (2)	0.005 (3)
C3	0.084 (5)	0.056 (3)	0.076 (4)	0.002 (3)	0.028 (3)	−0.005 (3)
C4	0.106 (5)	0.057 (3)	0.059 (3)	−0.010 (3)	0.029 (3)	−0.006 (3)
C5	0.097 (5)	0.078 (4)	0.058 (3)	−0.006 (4)	0.002 (3)	0.000 (3)
C6	0.066 (4)	0.069 (4)	0.062 (3)	0.000 (3)	0.002 (3)	−0.001 (3)
C7	0.035 (3)	0.052 (3)	0.060 (3)	−0.006 (2)	0.003 (2)	0.002 (2)
C8	0.037 (3)	0.055 (3)	0.054 (3)	−0.003 (2)	−0.0004 (19)	0.004 (2)
C9	0.044 (3)	0.058 (3)	0.056 (3)	−0.003 (2)	0.007 (2)	0.001 (2)
C10	0.048 (3)	0.103 (5)	0.048 (3)	−0.003 (3)	−0.007 (2)	0.002 (3)
C11	0.066 (4)	0.097 (4)	0.036 (3)	−0.003 (3)	0.005 (2)	0.002 (3)
C12	0.062 (4)	0.078 (4)	0.044 (3)	0.005 (3)	0.012 (2)	0.008 (2)
C13	0.058 (3)	0.078 (4)	0.037 (2)	0.013 (3)	0.002 (2)	−0.002 (2)
C14	0.057 (3)	0.062 (3)	0.042 (3)	−0.002 (3)	0.004 (2)	−0.001 (2)
C15	0.074 (4)	0.069 (4)	0.053 (3)	0.006 (3)	−0.002 (2)	0.019 (3)
C16	0.128 (7)	0.137 (7)	0.048 (3)	0.004 (6)	0.004 (4)	0.024 (4)
C17	0.127 (7)	0.081 (5)	0.087 (5)	−0.020 (5)	−0.011 (4)	0.022 (4)
C18	0.074 (5)	0.083 (5)	0.093 (4)	0.003 (3)	−0.012 (3)	0.022 (4)
O4	0.052 (2)	0.094 (3)	0.0467 (18)	0.017 (2)	0.0005 (14)	0.0030 (18)
O5	0.079 (3)	0.100 (3)	0.049 (2)	0.026 (2)	0.0150 (19)	0.003 (2)
O6	0.072 (3)	0.090 (3)	0.0415 (18)	0.026 (2)	0.0104 (16)	0.0123 (18)
N4	0.045 (3)	0.099 (4)	0.056 (3)	0.016 (2)	0.0039 (18)	0.004 (2)
N5	0.054 (3)	0.057 (2)	0.042 (2)	0.008 (2)	0.0049 (16)	0.0065 (17)
N6	0.073 (3)	0.067 (3)	0.0359 (19)	0.017 (2)	0.0124 (18)	0.0042 (19)
C19	0.042 (3)	0.053 (3)	0.053 (3)	−0.004 (2)	0.001 (2)	0.002 (2)
C20	0.055 (3)	0.067 (3)	0.061 (3)	0.006 (3)	0.007 (2)	0.003 (3)
C21	0.060 (4)	0.081 (4)	0.075 (4)	0.010 (3)	0.015 (3)	−0.005 (3)
C22	0.083 (5)	0.081 (4)	0.066 (4)	0.002 (3)	0.027 (3)	−0.007 (3)
C23	0.073 (4)	0.080 (4)	0.055 (3)	−0.008 (3)	0.006 (3)	0.001 (3)
C24	0.059 (3)	0.076 (4)	0.048 (3)	0.004 (3)	0.003 (2)	0.004 (3)
C25	0.048 (3)	0.050 (3)	0.046 (3)	−0.003 (2)	0.0002 (19)	0.005 (2)
C26	0.050 (3)	0.051 (3)	0.046 (2)	0.006 (2)	−0.0001 (19)	0.001 (2)
C27	0.060 (3)	0.055 (3)	0.038 (2)	0.007 (2)	0.005 (2)	0.001 (2)
C28	0.071 (4)	0.071 (3)	0.044 (3)	0.010 (3)	0.005 (2)	0.006 (2)
C29	0.089 (4)	0.083 (4)	0.037 (3)	0.014 (3)	0.014 (3)	0.014 (2)
C30	0.084 (4)	0.070 (3)	0.040 (3)	0.007 (3)	0.015 (2)	0.002 (2)
C31	0.064 (4)	0.065 (3)	0.043 (3)	0.011 (2)	0.013 (2)	0.009 (2)
C32	0.070 (4)	0.059 (3)	0.042 (3)	0.009 (3)	0.010 (2)	0.000 (2)
C33	0.069 (4)	0.071 (3)	0.042 (3)	0.006 (3)	−0.003 (2)	0.006 (2)
C34	0.108 (6)	0.076 (4)	0.075 (4)	−0.009 (4)	0.000 (4)	0.008 (3)
C35	0.086 (5)	0.111 (6)	0.048 (3)	0.005 (4)	0.004 (3)	0.007 (3)
C36	0.072 (5)	0.092 (5)	0.073 (4)	−0.002 (3)	−0.006 (3)	0.017 (3)

### Geometric parameters (Å, °)

**Table:**

O1—N2	1.417 (6)	C13—H13A	0.9700
O1—C8	1.349 (6)	C13—H13B	0.9700
O2—C14	1.309 (6)	C16—H16A	0.9600
O2—C15	1.487 (7)	C16—H16B	0.9600
O3—C14	1.233 (7)	C16—H16C	0.9600
O4—C26	1.347 (6)	C17—H17C	0.9600
O4—N4	1.416 (6)	C17—H17A	0.9600
O5—C32	1.235 (7)	C17—H17B	0.9600
O6—C33	1.477 (7)	C18—H18B	0.9600
O6—C32	1.319 (6)	C18—H18C	0.9600
N1—C7	1.385 (6)	C18—H18A	0.9600
N1—C8	1.278 (6)	C19—C20	1.375 (8)
N2—C7	1.312 (6)	C19—C24	1.411 (8)
N3—C13	1.462 (7)	C19—C25	1.446 (7)
N3—C14	1.357 (7)	C20—C21	1.379 (8)
N3—C12	1.448 (6)	C21—C22	1.386 (8)
N4—C25	1.309 (6)	C22—C23	1.351 (10)
N5—C25	1.372 (7)	C23—C24	1.377 (8)
N5—C26	1.278 (6)	C26—C27	1.501 (8)
N6—C31	1.465 (7)	C27—C28	1.515 (8)
N6—C32	1.349 (7)	C27—C31	1.511 (8)
N6—C30	1.449 (6)	C28—C29	1.515 (9)
C1—C7	1.447 (7)	C29—C30	1.503 (9)
C1—C2	1.382 (8)	C33—C34	1.503 (10)
C1—C6	1.392 (8)	C33—C35	1.515 (8)
C2—C3	1.382 (8)	C33—C36	1.497 (9)
C3—C4	1.376 (8)	C20—H20A	0.9300
C4—C5	1.379 (10)	C21—H21A	0.9300
C5—C6	1.375 (8)	C22—H22A	0.9300
C8—C9	1.481 (7)	C23—H23A	0.9300
C9—C13	1.516 (7)	C24—H24A	0.9300
C9—C10	1.499 (8)	C27—H27A	0.9800
C10—C11	1.527 (9)	C28—H28A	0.9700
C11—C12	1.497 (8)	C28—H28B	0.9700
C15—C17	1.516 (10)	C29—H29A	0.9700
C15—C16	1.508 (9)	C29—H29B	0.9700
C15—C18	1.476 (10)	C30—H30A	0.9700
C2—H2A	0.9300	C30—H30B	0.9700
C3—H3A	0.9300	C31—H31A	0.9700
C4—H4A	0.9300	C31—H31B	0.9700
C5—H5A	0.9300	C34—H34A	0.9600
C6—H6A	0.9300	C34—H34B	0.9600
C9—H9A	0.9800	C34—H34C	0.9600
C10—H10A	0.9700	C35—H35A	0.9600
C10—H10B	0.9700	C35—H35B	0.9600
C11—H11A	0.9700	C35—H35C	0.9600
C11—H11B	0.9700	C36—H36A	0.9600
C12—H12B	0.9700	C36—H36B	0.9600
C12—H12A	0.9700	C36—H36C	0.9600
			
N2—O1—C8	105.9 (3)	H17B—C17—H17C	110.00
C14—O2—C15	121.5 (5)	C15—C18—H18A	109.00
N4—O4—C26	105.9 (4)	C15—C18—H18B	109.00
C32—O6—C33	123.1 (5)	C15—C18—H18C	109.00
C7—N1—C8	104.4 (4)	H18A—C18—H18B	110.00
O1—N2—C7	104.0 (4)	H18A—C18—H18C	109.00
C12—N3—C14	121.8 (5)	H18B—C18—H18C	109.00
C13—N3—C14	122.9 (4)	C20—C19—C24	118.6 (5)
C12—N3—C13	114.9 (4)	C20—C19—C25	121.8 (5)
O4—N4—C25	103.3 (4)	C24—C19—C25	119.6 (5)
C25—N5—C26	103.8 (4)	C19—C20—C21	120.6 (5)
C30—N6—C32	118.9 (5)	C20—C21—C22	119.8 (6)
C31—N6—C32	121.1 (4)	C21—C22—C23	120.3 (5)
C30—N6—C31	116.1 (4)	C22—C23—C24	120.7 (5)
C2—C1—C6	119.0 (5)	C19—C24—C23	119.9 (5)
C2—C1—C7	120.5 (5)	N4—C25—N5	113.8 (4)
C6—C1—C7	120.4 (5)	N4—C25—C19	122.0 (5)
C1—C2—C3	120.5 (5)	N5—C25—C19	124.2 (4)
C2—C3—C4	120.1 (6)	O4—C26—N5	113.2 (5)
C3—C4—C5	119.8 (5)	O4—C26—C27	118.5 (4)
C4—C5—C6	120.4 (5)	N5—C26—C27	128.3 (5)
C1—C6—C5	120.2 (6)	C26—C27—C28	114.4 (5)
N2—C7—C1	122.8 (4)	C26—C27—C31	109.3 (4)
N1—C7—N2	112.7 (4)	C28—C27—C31	112.0 (4)
N1—C7—C1	124.6 (4)	C27—C28—C29	108.5 (5)
O1—C8—N1	113.0 (4)	C28—C29—C30	112.6 (5)
O1—C8—C9	118.3 (4)	N6—C30—C29	111.6 (5)
N1—C8—C9	128.8 (5)	N6—C31—C27	109.7 (4)
C8—C9—C10	115.4 (5)	O5—C32—O6	124.9 (5)
C8—C9—C13	108.1 (4)	O5—C32—N6	122.3 (5)
C10—C9—C13	111.3 (4)	O6—C32—N6	112.8 (5)
C9—C10—C11	110.4 (5)	O6—C33—C34	109.3 (5)
C10—C11—C12	111.4 (5)	O6—C33—C35	101.5 (5)
N3—C12—C11	110.3 (5)	O6—C33—C36	111.2 (5)
N3—C13—C9	110.9 (4)	C34—C33—C35	110.3 (5)
O3—C14—N3	121.4 (5)	C34—C33—C36	113.3 (6)
O2—C14—N3	112.2 (5)	C35—C33—C36	110.6 (5)
O2—C14—O3	126.3 (5)	C19—C20—H20A	120.00
C16—C15—C17	111.1 (6)	C21—C20—H20A	120.00
O2—C15—C18	111.1 (5)	C20—C21—H21A	120.00
O2—C15—C17	109.1 (5)	C22—C21—H21A	120.00
O2—C15—C16	100.8 (5)	C21—C22—H22A	120.00
C16—C15—C18	111.5 (6)	C23—C22—H22A	120.00
C17—C15—C18	112.6 (6)	C22—C23—H23A	120.00
C1—C2—H2A	120.00	C24—C23—H23A	120.00
C3—C2—H2A	120.00	C19—C24—H24A	120.00
C2—C3—H3A	120.00	C23—C24—H24A	120.00
C4—C3—H3A	120.00	C26—C27—H27A	107.00
C3—C4—H4A	120.00	C28—C27—H27A	107.00
C5—C4—H4A	120.00	C31—C27—H27A	107.00
C6—C5—H5A	120.00	C27—C28—H28A	110.00
C4—C5—H5A	120.00	C27—C28—H28B	110.00
C1—C6—H6A	120.00	C29—C28—H28A	110.00
C5—C6—H6A	120.00	C29—C28—H28B	110.00
C13—C9—H9A	107.00	H28A—C28—H28B	108.00
C8—C9—H9A	107.00	C28—C29—H29A	109.00
C10—C9—H9A	107.00	C28—C29—H29B	109.00
C9—C10—H10A	110.00	C30—C29—H29A	109.00
C9—C10—H10B	110.00	C30—C29—H29B	109.00
C11—C10—H10A	110.00	H29A—C29—H29B	108.00
C11—C10—H10B	110.00	N6—C30—H30A	109.00
H10A—C10—H10B	108.00	N6—C30—H30B	109.00
C10—C11—H11A	109.00	C29—C30—H30A	109.00
C10—C11—H11B	109.00	C29—C30—H30B	109.00
C12—C11—H11B	109.00	H30A—C30—H30B	108.00
H11A—C11—H11B	108.00	N6—C31—H31A	110.00
C12—C11—H11A	109.00	N6—C31—H31B	110.00
H12A—C12—H12B	108.00	C27—C31—H31A	110.00
C11—C12—H12B	110.00	C27—C31—H31B	110.00
N3—C12—H12A	110.00	H31A—C31—H31B	108.00
N3—C12—H12B	110.00	C33—C34—H34A	109.00
C11—C12—H12A	110.00	C33—C34—H34B	110.00
N3—C13—H13A	109.00	C33—C34—H34C	110.00
N3—C13—H13B	110.00	H34A—C34—H34B	110.00
C9—C13—H13A	109.00	H34A—C34—H34C	109.00
C9—C13—H13B	109.00	H34B—C34—H34C	110.00
H13A—C13—H13B	108.00	C33—C35—H35A	110.00
C15—C16—H16A	109.00	C33—C35—H35B	109.00
C15—C16—H16B	109.00	C33—C35—H35C	109.00
C15—C16—H16C	110.00	H35A—C35—H35B	109.00
H16B—C16—H16C	110.00	H35A—C35—H35C	109.00
H16A—C16—H16B	109.00	H35B—C35—H35C	109.00
H16A—C16—H16C	109.00	C33—C36—H36A	110.00
H17A—C17—H17C	109.00	C33—C36—H36B	109.00
C15—C17—H17A	109.00	C33—C36—H36C	109.00
C15—C17—H17B	109.00	H36A—C36—H36B	110.00
C15—C17—H17C	109.00	H36A—C36—H36C	109.00
H17A—C17—H17B	109.00	H36B—C36—H36C	109.00
			
N2—O1—C8—N1	−1.4 (6)	C2—C1—C7—N2	20.5 (8)
N2—O1—C8—C9	178.5 (4)	C6—C1—C2—C3	2.0 (9)
C8—O1—N2—C7	−0.3 (5)	C7—C1—C2—C3	179.3 (5)
C15—O2—C14—O3	−1.6 (9)	C2—C1—C6—C5	−2.0 (9)
C15—O2—C14—N3	−178.6 (5)	C7—C1—C6—C5	−179.4 (5)
C14—O2—C15—C17	−63.1 (7)	C2—C1—C7—N1	−158.8 (5)
C14—O2—C15—C18	61.6 (7)	C6—C1—C7—N2	−162.2 (5)
C14—O2—C15—C16	179.9 (6)	C6—C1—C7—N1	18.5 (8)
N4—O4—C26—C27	176.2 (4)	C1—C2—C3—C4	−1.1 (9)
N4—O4—C26—N5	−2.2 (6)	C2—C3—C4—C5	0.3 (10)
C26—O4—N4—C25	2.7 (6)	C3—C4—C5—C6	−0.4 (10)
C33—O6—C32—N6	−171.1 (5)	C4—C5—C6—C1	1.3 (9)
C32—O6—C33—C36	−65.0 (7)	N1—C8—C9—C10	−179.6 (5)
C32—O6—C33—C34	60.9 (7)	O1—C8—C9—C13	125.9 (5)
C33—O6—C32—O5	10.3 (9)	O1—C8—C9—C10	0.6 (7)
C32—O6—C33—C35	177.4 (5)	N1—C8—C9—C13	−54.3 (7)
C7—N1—C8—O1	2.3 (6)	C8—C9—C10—C11	177.3 (5)
C8—N1—C7—N2	−2.5 (6)	C13—C9—C10—C11	53.6 (7)
C8—N1—C7—C1	176.8 (5)	C8—C9—C13—N3	179.4 (4)
C7—N1—C8—C9	−177.6 (5)	C10—C9—C13—N3	−52.9 (7)
O1—N2—C7—N1	1.7 (5)	C9—C10—C11—C12	−55.1 (7)
O1—N2—C7—C1	−177.7 (4)	C10—C11—C12—N3	54.9 (7)
C13—N3—C14—O3	178.0 (5)	C24—C19—C20—C21	−2.0 (8)
C13—N3—C12—C11	−55.9 (7)	C25—C19—C20—C21	179.1 (5)
C14—N3—C12—C11	117.0 (6)	C20—C19—C24—C23	1.8 (8)
C12—N3—C13—C9	54.8 (6)	C25—C19—C24—C23	−179.3 (5)
C14—N3—C13—C9	−118.0 (5)	C20—C19—C25—N4	−10.0 (8)
C12—N3—C14—O2	−177.1 (5)	C20—C19—C25—N5	173.9 (5)
C12—N3—C14—O3	5.7 (8)	C24—C19—C25—N4	171.1 (5)
C13—N3—C14—O2	−4.8 (7)	C24—C19—C25—N5	−4.9 (8)
O4—N4—C25—N5	−2.4 (6)	C19—C20—C21—C22	2.1 (9)
O4—N4—C25—C19	−178.8 (4)	C20—C21—C22—C23	−2.0 (10)
C26—N5—C25—C19	177.4 (5)	C21—C22—C23—C24	1.8 (10)
C25—N5—C26—O4	0.8 (6)	C22—C23—C24—C19	−1.7 (9)
C26—N5—C25—N4	1.1 (6)	O4—C26—C27—C28	15.2 (7)
C25—N5—C26—C27	−177.4 (5)	O4—C26—C27—C31	141.7 (5)
C32—N6—C30—C29	150.6 (5)	N5—C26—C27—C28	−166.7 (5)
C31—N6—C30—C29	−51.5 (7)	N5—C26—C27—C31	−40.2 (7)
C32—N6—C31—C27	−149.7 (5)	C26—C27—C28—C29	−178.1 (4)
C30—N6—C32—O5	−10.6 (9)	C31—C27—C28—C29	56.8 (6)
C30—N6—C32—O6	170.8 (5)	C26—C27—C31—N6	176.9 (4)
C31—N6—C32—O5	−167.4 (6)	C28—C27—C31—N6	−55.3 (6)
C31—N6—C32—O6	14.0 (8)	C27—C28—C29—C30	−54.6 (6)
C30—N6—C31—C27	52.9 (6)	C28—C29—C30—N6	51.8 (7)

### Hydrogen-bond geometry (Å, °)

**Table:**

*D*—H···*A*	*D*—H	H···*A*	*D*···*A*	*D*—H···*A*
C13—H13A···N2^i^	0.97	2.61	3.352 (7)	133
C18—H18A···N1^i^	0.96	2.61	3.490 (9)	153

Symmetry codes: (i) *x*+1, *y*, *z*.
